# Association of systemic inflammation index with psoriasis risk and psoriasis severity: A retrospective cohort study of NHANES 2009 to 2014

**DOI:** 10.1097/MD.0000000000037236

**Published:** 2024-02-23

**Authors:** Huan-huan Guo, Ruo-xi Chen

**Affiliations:** a Dermatology, Longyan First Affiliated Hospital of Fujian Medical University, Longyan 364000, China.

**Keywords:** biomarker, NHANES database, psoriasis, systemic inflammation index

## Abstract

To investigate the association of systemic inflammation index (SII) with psoriasis risk and psoriasis severity. This is a retrospective cohort study based on data from the National Health and Nutrition Examination Survey database from 2009 to 2014. The psoriasis information was obtained from the questionnaire data, and the SII was calculated as neutrophil × platelet/lymphocyte. We performed matching by controlling age and gender to reach a 1:2 ratio for better statistical power. Weighted logistic regression analysis, subgroup analysis, restricted cubic spline analysis, and threshold analysis were used to evaluate the association of SII with psoriasis risk. Besides, mediation analysis was conducted to assess the possible regulatory path. Finally, the receiver operating characteristic curve was plotted to analyze the predictive value of SII for psoriasis severity. The study involved 16,466 participants including 16,020 no-psoriasis participants and 446 psoriasis participants. After matching, psoriasis and non-psoriasis individuals were 446 and 892, respectively. SII was significantly higher in the psoriasis group than the non-psoriasis group (*P* < .05). Additionally, white blood cells and monocytes were significantly linked to psoriasis risk and SII scores (*P* < .05). Besides, SII elevation was an independent predictor for upregulated psoriasis risk (*P* < .05). There was a nonlinear relationship between SII and psoriasis risk (*P* nonlinear < .05), which was not mediated by white blood cells and monocytes. Unexpectedly, SII had no significance in predicting SII severity (*P* > .05). SII can independently predict psoriasis risk but has no impact on psoriasis severity. Further, SII serves as a potential and robust biomarker for identifying high-risk psoriasis individuals.

## 1. Introduction

Psoriasis is a chronic and immune-mediated skin disease, which is characterized by the formation of scaly, indurated, erythematous plaques.^[1]^ Psoriasis occurs in approximately 125 million people worldwide with a global incidence of 2% to 3% but varies according to regions. The morbidity ranges from 0.5% in parts of Asia and some African populations to as high as 11% in Scandinavian and Caucasian populations^[2–4]^ Epidermal hyperplasia, dilation, conspicuous blood vessels in the dermis, and inflammatory leucocyte infiltrations are the main histologic characteristics of psoriasis.^[5]^ Multiple factors such as genetic and environmental factors, systemic corticosteroid withdrawal, and oxidative stress contributed to psoriasis.^[6,7]^ Although it is a nonfatal chronic disease, psoriasis not solely affects the skin, but also the joints and is associated with an increased risk of other diseases. Psoriasis patients are at risk of developing stroke, myocardial infarction, type 2 diabetes, obesity, and hypertension.^[8,9]^ Besides, psoriasis patients are susceptible to suicidality, anxiety, and depression, leading to adverse effects on the quality of their lives and bringing huge physical and psychological burdens.^[10]^ Therefore, it is necessary to identify modifiable risk factors and take early interventions for psoriasis prevention and treatment.

The complete blood count (CBC) is a routine clinical checkup and the related indices such as mean platelet volume (MPV) and red cell distribution width (RDW) have been used as prognostic biomarkers for numerous diseases.^[11–13]^ The systemic inflammation index (SII) is a novel CBC indicator that reflects the systemic immune and inflammatory status of the human body.^[14]^ SII is calculated based on the levels of neutrophils, platelets, and lymphocytes.^[15]^ Previous studies have revealed SII as an evaluable predictor for disease risk and prognosis assessment, particularly in the field of cancer. Chen et al^[16]^ reported that high SII is a powerful tool to predict shorter overall survival and disease-free survival in patients with colorectal cancer. Besides, high SII is significantly related to diminished survival in patients with gastroesophageal adenocarcinomas.^[17]^ Moreover, an upregulated level of SII was observed in those with rheumatoid arthritis.^[18]^ Sporadic studies have demonstrated that SII might serve as a potential biomarker in the development of psoriasis and psoriatic arthritis^[19,20]^; however, lack of systemic research investigating the association of SII with psoriasis risk and psoriasis severity.

Herein, this study aimed to determine the association of SII with psoriasis risk and psoriasis severity in adults aged ≥ 18 years using a large sample of participants from the National Health and Nutrition Examination Survey (NHANES) database.

## 2. Materials and methods

### 2.1. Study population

This is a retrospective study and all subject information was extracted from the NHANES database. Data from the NHANES database is collected biennially. Detailed methods can be extracted from the official website (http://www.cdc.gov/nchs/nhanes.htm, accessed on November 1, 2022). All the protocols were approved by the National Center for Health Statistics Institutional Review Board, and informed consent was required from participants. Data for psoriasis were available in the 2009 to 2014 cycles. The inclusion criteria included: age ≥ 18 years; data for psoriasis; with complete SII data; with complete data for covariates. The exclusion criteria included: age <18 years; missing data for psoriasis, SII, or covariates; extreme data for SII or covariates. Finally, 16,020 non-psoriasis and 446 psoriasis individuals were selected for this study. For better statistical power, we conducted matching according to age and gender to adjust the baseline characteristics of the 2 groups, maintaining a ratio of 1:2 for cases, resulting in a final study sample of psoriasis (*n* = 446) and non-psoriasis (*n* = 892).

### 2.2. Outcome measures

Psoriasis risk was the primary outcome measure, which was determined by affirmative responses to the question “Have you ever been told by a doctor or other healthcare professional that you had psoriasis?” Participants were divided into 2 groups: the no-psoriasis group (*n* = 16,020) and the psoriasis group (*n* = 446). Psoriasis severity was the secondary outcome measure, which was determined by a questionnaire in the 2011 to 2014 circles. Participants were asked if they had little or no-psoriasis (<1 palm of hand), only a few patches (1 or 2 palms of hand), scattered patches (3–10 palms of hand), and extensive patches (>10 palms of hand). Participants were categorized into 2 groups based on psoriasis severity: no or mild group (no/little/few) and moderate to severe group (scattered/extensive) with 227 and 52 participants in each group, respectively.

### 2.3. SII and covariates

SII was calculated based on the results of the CBC test in the NHANES database. Neutrophil count, platelet count, and lymphocyte count were measured in 1000 cells/µL. The formula was as follows: SII = neutrophil × platelet/lymphocyte.^[21]^ The other covariates were also obtained from this database: age, gender (female and male), body mass index (BMI), race (Hispanic, White, Black, and other races), marital status (married/partner, widowed/divorced/separated), household income (<20,000, 20,000–74,999, ≥75,000), and the CBC including monocyte, white blood cell (WBC) count, RDW, red blood cell (RBC) count, hemoglobin, hematocrit, mean cell volume (MCV), mean cell hemoglobin (MCH), mean corpuscular hemoglobin concentration and MPV.

### 2.4. Statistical analysis

Statistical analyses were performed using SPSS software version 2.0 and Empower Stats version 4.1. SII was categorized into quartiles: Q1 (<333.86), Q2 (333.86–472.50), Q3 (473.26–664.36), and Q4 (>667.89). The categorical variables were expressed as count (percent) and the differences between the groups were compared by Chi-square or Fisher’s exact tests. Continuous variables were presented as median (interquartile range) (skewed distribution). The differences between participants with or without psoriasis and the differences among those grouped by SII quartiles were evaluated using the Mann–Whitney *U* test and Kruskal–Wallis test, respectively. A *P* value <.05 was considered statistically significant.

The association of SII with psoriasis was examined by logistic regression analysis using 3 models. Model 1: no covariate was adjusted; Model 2: age, gender, BMI, race, marital status, and household income were adjusted; Model 3: all covariates including age, gender, BMI, race, marital status, household income, monocyte count, WBC count, RDW, RBC count, hemoglobin, hematocrit, MCV, MCH, and MPV were adjusted. Then, stratified analysis was performed to evaluate the correlation between SII and psoriasis risk in different subgroups according to age (<60 years/≥60 years), gender (female/male), and race (Hispanic/White/Black/Other races). Using restricted cubic spline (RCS) analysis, the nonlinear relationship between SII and psoriasis was assessed. If there is nonlinearity, threshold analysis was performed to investigate the association and inflection points between SII and psoriasis status. Mediation analysis was performed to examine the potential regulatory path. Finally, receiver operating characteristic curve analysis was performed and the area under the curve was calculated to investigate the value of SII in predicting psoriasis severity.

## 3. Results

### 3.1. Baseline characteristics of participants

A total of 16,466 participants (16,020 non-psoriasis and 446 psoriasis individuals) met the inclusion and exclusion criteria from the NHANES database. There were differences in several biographical characteristics between the non-psoriasis and psoriasis groups (Table 1). Before matching, participants in the psoriasis group were more likely to be older and White, and they had significantly higher BMI than those in the non-psoriasis group (*P* < .05). The psoriasis group had a significantly higher proportion of “Widowed/divorced/separated” (27.1% vs 21.9%), but a lower proportion of “Never married” (15.3% vs 19.1%) (*P* < .05). After matching, there were no significant differences in biographical characteristics except in race between the 2 groups.

**Table 1:** Biographical characteristics of the study population based on psoriasis.

Characteristics	Before matching			After matching		
	Psoriasis (*n* = 446)	Non-psoriasis (*n* = 1620)	*P* value	Psoriasis (*n* = 446)	Non-psoriasis (*n* = 892)	*P* value
Age, years	52.00 (39.00, 64.00)	47.00 (31.00, 62.00)	<.001	52.00 (39.00, 64.00)	51.00 (38.00, 63.00)	.211
Gender, %						
Male	213 (47.8)	7806 (48.7)	.701	213 (47.8)	401 (45.0)	.352
Female	233 (52.2)	8214 (51.3)		233 (52.2)	491 (55.0)	
Body mass index, kg/m^2^	28.50 (25.30, 33.60)	27.70 (24.00, 32.30)	<.001	28.50 (25.30, 33.60)	28.10 (24.49, 32.90)	.112
Race, %						
Hispanic	81 (18.2)	3959 (24.7)	<.001	81 (18.2)	232 (26.0)	<.001
White	259 (58.1)	6793 (42.4)		259 (58.1)	369 (41.4)	
Black	49 (11.0)	3357 (21.0)		49 (11.0)	196 (22.0)	
Others	57 (12.8)	1911 (11.9)		57 (12.8)	95 (10.7)	
Marital status, %						
Married/partner	253 (57.6)	8965 (59.0)	.013	253 (57.6)	510 (58.8)	.820
Widowed/divorced/separated	119 (27.1)	3335 (21.9)		119 (27.1)	221 (25.5)	
Never married	67 (15.3)	2906 (19.1)		67 (15.3)	136 (15.7)	
Household income, $						
<20,000	108 (24.9)	3394 (22.3)	.365	108 (24.9)	178 (21.0)	.251
20,000–74,999	209 (49.2)	7943 (52.1)		209 (49.2)	449 (52.9)	
≥75,000	110 (25.9)	3909 (25.6)		110 (25.9)	221 (26.1)	

As for laboratory variables, monocyte count, WBC count, and RDW in the psoriasis group were significantly higher than those in the non-psoriasis group (all *P* < .05). Other covariates such as RBC count, hemoglobin, hematocrit, MCV, MCH, mean corpuscular hemoglobin concentration, and MPV were not significantly different from the 2 groups. After matching, the psoriasis group presented a higher monocyte count, and WBC count (all *P* < .05) (Table 2).

**Table 2:** Laboratory variables of the study population based on psoriasis.

Characteristics	Before matching			After matching		
	Psoriasis (*n* = 446)	Non-psoriasis (*n* = 1620)	*P* value	Psoriasis (*n* = 446)	Non-psoriasis (*n* = 892)	*P* value
Monocyte count, 10^3^/µL	0.50 (0.50, 0.70)	0.50 (0.40, 0.60)	<.001	0.50 (0.50, 0.70)	0.50 (0.40, 0.60)	.007
White blood cell count, 10^3^/µL	7.10 (5.80, 8.80)	6.90 (5.60, 8.30)	.014	7.10 (5.80, 8.80)	6.80 (5.70, 8.10)	.029
Red cell distribution width, %	13.10 (12.50, 13.90)	13.00 (12.40, 13.70)	.034	13.10 (12.50, 13.90)	13.00 (12.50, 13.70)	.429
Red blood cell count, million cells/µL	4.59 (4.25, 4.95)	4.60 (4.28, 4.95)	.430	4.59 (4.25, 4.95)	4.60 (4.26, 4.91)	.925
Hemoglobin, g/dL	14.00 (13.00, 15.00)	14.00 (13.10, 15.00)	.770	14.00 (13.00, 15.00)	13.90 (13.00, 15.00)	.807
Hematocrit, %	41.00 (38.30, 44.20)	41.20 (38.30, 44.10)	.765	41.00 (38.30, 44.20)	41.00 (38.30, 43.80)	.910
Mean cell volume, fL	89.80 (87.00, 93.00)	89.80 (86.40, 92.90)	.701	89.80 (87.00, 93.00)	89.80 (86.60, 92.60)	.742
Mean cell hemoglobin, pg	30.70 (29.30, 31.90)	30.70 (29.30, 31.80)	.847	30.70 (29.30, 31.90)	30.70 (29.20, 31.80)	.763
Mean corpuscular hemoglobin concentration, g/dL	34.00 (33.40, 34.70)	34.10 (33.40, 34.70)	.906	34.00 (33.40, 34.70)	34.00 (33.40, 34.70)	.902
Mean platelet volume, fL	8.10 (7.60, 8.80)	8.10 (7.60, 8.80)	.735	8.10 (7.60, 8.80)	8.20 (7.60, 8.90)	.866

SII is a parameter obtained by calculating the ratio between (neutrophils × platelets) and lymphocytes. Data on neutrophils, platelets, lymphocytes, and SII are shown in Table 3. The psoriasis group exhibited remarkably higher neutrophils and SII, but lower lymphocytes than the non-psoriasis group (*P* < .05).

**Table 3:** Immune cell count data.

Characteristics	Before matching			After matching		
	Psoriasis (*n* = 446)	Non-psoriasis (*n* = 1620)	*P* value	Psoriasis (*n* = 446)	Non-psoriasis (*n* = 892)	*P* value
Neutrophils, 10^3^/µL	4.30 (3.30, 5.50)	3.90 (3.10, 5.10)	<.001	4.30 (3.30, 5.50)	4.00 (3.00, 4.90)	<.001
Platelets, 10^3^/µL	231.00 (196.00, 271.00)	231.00 (196.00, 273.00)	.701	231.00 (196.00, 271.00)	233.00 (200.00, 276.00)	.324
Lymphocytes, 10^3^/µL	1.90 (1.50, 2.40)	2.00 (1.60, 2.50)	<.001	1.90 (1.50, 2.40)	2.10 (1.60, 2.50)	<.001
Systemic inflammation index	514.86 (364.11, 742.61)	446.73 (318.93, 632.32)	<.001	514.86 (364.11, 742.61)	452.25 (320.60, 624.25)	<.001

Systemic inflammation index = neutrophil × platelet/lymphocyte

The clinical characteristics of the individuals based on the quartiles of SII are exhibited in Table S1, Supplemental Digital Content, http://links.lww.com/MD/L647. There was statistical significance in terms of race, monocyte count, WBC count, RDW, RBC count, hemoglobin, hematocrit, MPV, and psoriasis status among the 4 SII groups. Participants in the Quartile 3 and Quartile 4 groups tended to be white; they had elevated monocyte count, WBC count, and RDW while having lower RBC count, hemoglobin, hematocrit, and MPV (*P* < .05).

### 3.2. Correlation between SII and psoriasis risk

The association results of SII with psoriasis risk are demonstrated in Table 4. When no covariate was adjusted in Model 1, SII was significantly related to psoriasis risk (*P* < .05). After adjusting age, gender, BMI, race, marital status, and household income in Model 2, higher SII was associated with an increased risk of psoriasis (*P* < .05). After adjusting all the covariates in Model 3, SII remained an independent risk factor for psoriasis (*P* < .05). Sensitivity analysis was performed with SII quartiles and the ORs for Q4 in Model 1, Model 2, and Model 3 were 1.902, 1.858, and 2.240, respectively, compared to Quartile 1. The trend test showed that participants with higher SII had an increased risk of psoriasis (*P* for trend < .05).

**Table 4:** Association of SII with psoriasis using weighted logistic regression analysis.

	Model 1		Model 2		Model 3	
	OR (95% CI)	*P* value	OR (95% CI)	*P* value	OR (95% CI)	*P* value
SII,	2.906 (1.742–4.847)	<.001	2.701 (1.589–4.593)	<.001	3.782 (1.994–7.176)	<.001
SII quartiles						
Q1	Reference		Reference		Reference	
Q2	0.911 (0.649–1.277)	.588	0.883 (0.623–1.253)	.486	0.936 (0.655–1.336)	.715
Q3	1.296 (0.934–1.797)	.120	1.245 (0.885–1.752)	.209	1.375 (0.958–1.973)	.084
Q4	1.902 (1.380–2.622)	<.001	1.858 (1.328–2.601)	<.001	2.240 (1.523–3.295)	<.001
P for trend		<.001		<.001		<.001

95% CI = 95% confidence interval, OR = odds ratio, SII = systemic inflammation index

**Figure 1.**
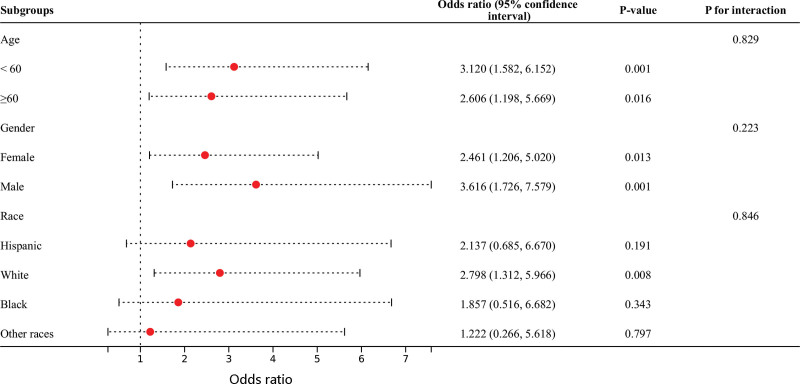
The forest plot revealed the significant relationship between systemic inflammation index (SII) and psoriasis risk stratified by age, gender, and race.

Further, subgroup analysis demonstrated that SII was significantly linked to psoriasis status regardless of age and gender. Besides, a notable correlation between SII and psoriasis status among “White” (all *P* < .05) (Fig. 1). Interaction tests revealed that age, gender, and race were not interactive factors in the relationship between SII and psoriasis (*P* for interaction > .05).

**Figure 2.**
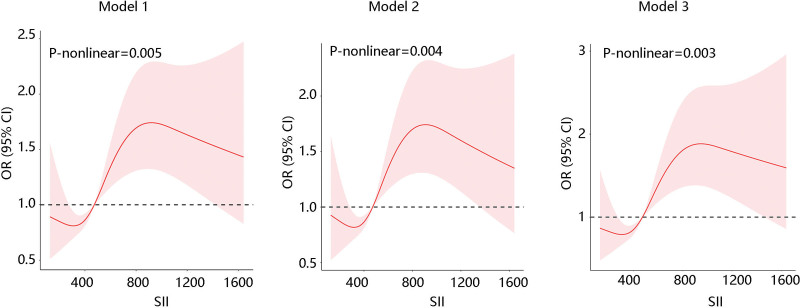
Nonlinear relationship between SII and psoriasis risk in 3 models using restricted cubic spline analysis. Model 1: No covariate was adjusted; Model 2: age, gender, BMI, race, marital status, and household income were adjusted; Model 3: all covariates including age, gender, BMI, race, marital status, household income, monocyte count, WBC count, RDW, RBC count, hemoglobin, hematocrit, MCV, MCH, and MPV were adjusted.

Moreover, RCS analysis results revealed a nonlinear correlation between SII and psoriasis in 3 models (*P* for nonlinearity > .05) (Fig. 2). Adjusted variables: age, gender, BMI, race, marital status, household income, monocyte count, WBC count, RDW, RBC count, hemoglobin, hematocrit, MCV, MCH, and MPV. The inflection point was 792. On the left side of the inflection point, the association was not significant (*P* > .05). On the right side of the inflection point, the psoriasis risk was upregulated with the increase of SII (*P* < .05) (Table 5).

**Table 5:** Threshold effect analysis of SII on psoriasis using piece-wise linear regression models.

	Adjusted odds ratio (95% confidence interval)	*P* value
SII		
Inflection point	792	
SII < 792	0.8 (0.1, 4.7)	.833
SII ≥ 792	4.0 (1.7, 9.1)	.001
Log likelihood ratio		.006

SII = systemic inflammation index

### 3.3. Mediation analysis

**Figure 3.**
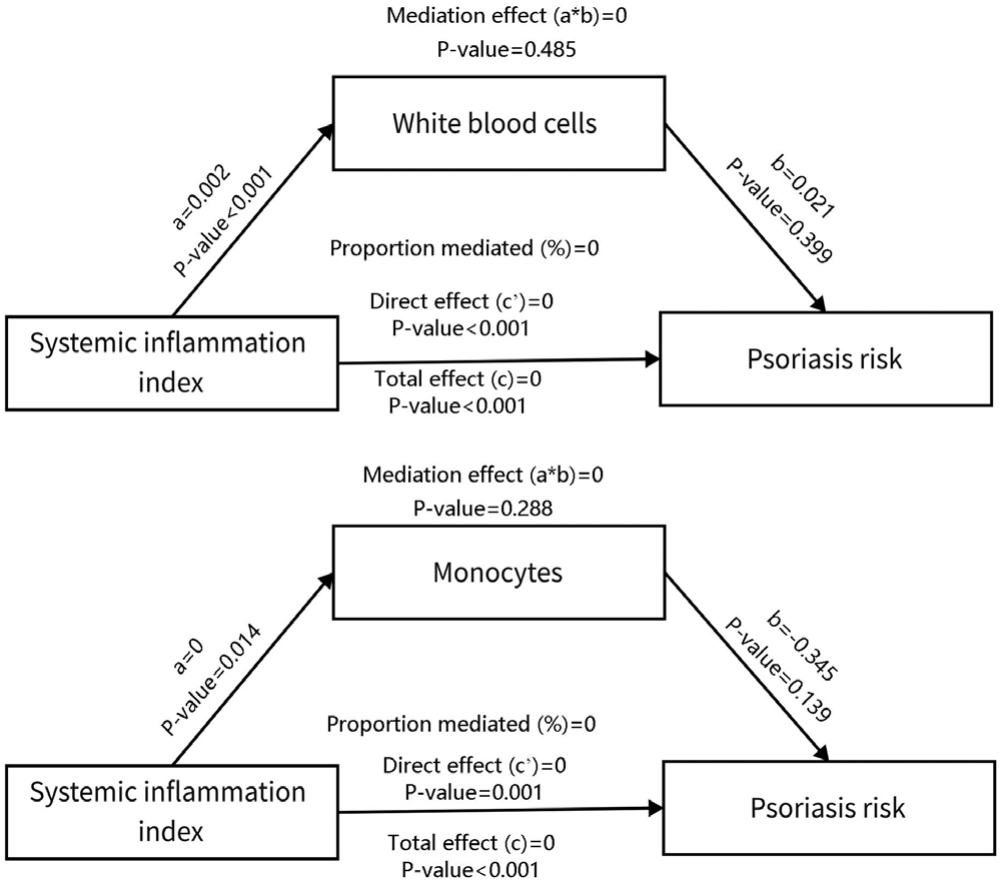
Mediation analysis showing the regulatory path of SII on psoriasis risk. The mediation effect = zero indicated that the white blood cells and monocytes did not play mediating role in the association of SII with psoriasis risk.

We have demonstrated that monocyte and WBC were significantly correlated with SII and psoriasis risk. Therefore, we performed a mediation analysis to investigate the regulatory path. Surprisingly, monocyte and WBC did not play a significant mediating role in the association of SII with psoriasis risk (*P* > .05) (Fig. 3).

### 3.4. Correlation between SII and psoriasis severity

Due to the significant role of SII in predicting psoriasis risk, we further elucidated the value of SII in predicting psoriasis severity. Participants in the no or mild group and moderate to severe group were 227 and 52, respectively. The distribution of various covariates in different groups is shown in Table S2, Supplemental Digital Content, http://links.lww.com/MD/L648. Surprisingly, there was no significant difference in SII between the 2 groups. In addition, the receiver operating characteristic curve analysis revealed no obvious predictive ability of SII for psoriasis severity (area under the curve = 0.54) (Figure S1, Supplemental Digital Content, http://links.lww.com/MD/L646).

## 4. Discussion

This study found that SII had a positive and nonlinear correlation with psoriasis risk. This significant relationship still existed after adjusting all covariates. The subgroup analysis showed that SII elevation was notably related to higher psoriasis risk in all subgroups except Hispanics, Blacks, and other races. Moreover, when SII is higher than 792, the risk of psoriasis will increase significantly. Interestingly, WBC or monocytes did not mediate the association of SII with psoriasis. Whereas, we found that SII had insignificant value in predicting psoriasis severity.

First, we assessed the distributions of variables in different groups according to psoriasis status and SII quartiles. Compared with the non-psoriasis group, SII was upregulated in the psoriasis group with statistical significance. Besides, significant differences in race, monocyte, and WBC were observed in 2 psoriasis groups as well as 4 SII groups. Liu et al^[22]^ revealed that SII was positively related to rheumatoid arthritis risk. Additionally, SII served as a reliable inflammatory marker for predicting the osteoporosis risk or low bone mineral density among postmenopausal women.^[23]^ It has also been demonstrated that SII had a positive correlation with an increased risk of presenting hepatic steatosis, but not correlated with liver fibrosis.^[24,25]^ Moreover, SII was positively linked to the severity of psoriatic arthritis in a study including 106 psoriatic arthritis and 103 healthy participants.^[20]^ Similarly, Yorulmaz et al^[19]^ enrolled 171 patients and 171 controls in the study to exhibit higher SII in patients with moderate/severe psoriasis and those with psoriatic arthritis. Our study exhibited that the SII level of psoriasis patients was higher than that of non-psoriasis participants, but cannot reflect the psoriasis severity. The difference might be due to the subject selection bias.

Psoriasis is characterized by persistent inflammation that causes uncontrolled keratinocyte proliferation and dysfunctional differentiation. The histological appearance of psoriatic plaques is spinous layer hyperplasia, which is covered by an inflammatory infiltrate composed of neutrophils, dermal dendritic cells, T cells, and macrophages.^[26]^ Keratinocytes, plasmacytoid dendritic cells, macrophages, and natural killer T cells play essential roles in the initial stage of psoriasis, which can secrete cytokines to activate myeloid dendritic cells. Antimicrobial peptides are secreted by keratinocytes in response to injury and are characteristically overexpressed in psoriatic skin. The recognition of antimicrobial peptides including LL37 and S100 is the proposed mechanism of psoriasis progression.^[27]^ Damaged keratinocytes-released LL37 forms a complex with self-genetic material from other damaged cells. DNA-LL37 stimulates toll-like receptor 9 in plasmacytoid dendritic cells to secrete IFN-α, activating myeloid dendritic cells to secrete IL-23 and IL-12.^[28]^ IL-23 is essential for the survival and proliferation of Th17 and Th22 cells, further secreting TNF-α, IL-22, and IL-17. IL-12 induces the differentiation of naïve cells into Th1 cells, secreting TNF-α and IFN-γ.^[29,30]^ These cytokines result in the proliferation of keratinocytes, upregulation of endothelial adhesion molecules and angiogenic mediators, as well as elevated immune cell infiltrates, further worsening the psoriasis progression.^[30]^ Therefore, SII based on neutrophils, platelets, and lymphocytes might increase psoriasis risk via the involvement of immune cell response. Clinicians should take effective and timely interventions for those at high-risk of developing psoriasis according to the SII score.

Subsequently, we investigated whether WBC and monocytes play mediating roles in the association of SII with psoriasis risk since the 2 indicators were closely linked to both psoriasis and SII. We found that WBC or monocytes did not mediate the correlation between SII and psoriasis risk. Future studies are required to validate this finding.

For strengths, this is the first study to analyze the association of SII with psoriasis risk and psoriasis severity in a large-scale population using the NHANES database. In addition, we adopted 3 logistic regression models and adjusted other covariates to make results more reliable and accurate. Further, RCS and threshold analysis were employed to evaluate the nonlinear correlation between SII and psoriasis risk. For limitations, some classic inflammatory variables such as IL-12 and TNF-α which are not available in the NHANES database cannot be enrolled in the study to acquire more comprehensive results. Due to small samples with psoriasis severity information, the role of SII in psoriasis severity should be verified in a larger sample size in the future. However, our study still provides theoretical support for future research and clinical interventions. We will enroll more classic inflammatory markers in our own cohort to explore the association of SII with psoriasis risk and psoriasis severity in the future.

In conclusion, SII is significantly related to psoriasis risk but not the disease severity. Moreover, SII may be a cost-effective and easily accessible tool to predict the risk of psoriasis and provide references for clinicians to act appropriately for psoriasis prevention.

## Author contributions

**Conceptualization:** Huan-huan Guo.

**Data curation:** Huan-huan Guo.

**Methodology:** Huan-huan Guo, Ruo-xi Chen.

**Supervision:** Huan-huan Guo.

**Validation:** Huan-huan Guo.

**Writing—original draft:** Huan-huan Guo, Ruo-xi Chen.

**Writing—review & editing:** Huan-huan Guo.

**Formal analysis:** Ruo-xi Chen.

## Supplementary Material






