# Supplementary material for: Association of systemic inflammation index with psoriasis risk and psoriasis severity: A retrospective cohort study of NHANES 2009 to 2014
Source: Medicine (Baltimore). 2024 Feb 23;103(8):e37236. doi: 10.1097/MD.0000000000037236 (PMC11309634; doi:10.1097/MD.0000000000037236)
Supplement: Supplementary file 1 [file medi-103-e37236-s001.docx]

| **Table S1** Basic characteristics of the study population based on SII quartiles | | | | | |
| --- | --- | --- | --- | --- | --- |
|  |  | SII quartiles |  |  | P-value |
|  | Q1(< 333.86) (n=334) | Q2 (333.86-472.50) (n=335) | Q3 (473.26-664.36) (n=335) | Q4 (> 667.89) (n=334) |  |
| Age, years | 50.00 [38.00, 63.00] | 51.00 [36.00, 63.00] | 51.00 [39.00, 63.00] | 52.00 [38.00, 67.00] | 0.383 |
| Gender, % |  |  |  |  | 0.123 |
| Male | 171 (51.2) | 154 (46.0) | 146 (43.6) | 143 (42.8) |  |
| Female | 163 (48.8) | 181 (54.0) | 189 (56.4) | 191 (57.2) |  |
| BMI, kgm^2^ | 27.80 [24.50, 32.20] | 28.25 [24.30, 33.43] | 28.24 [24.80, 33.11] | 28.60 [25.10, 33.74] | 0.298 |
| Race, % |  |  |  |  | <0.001 |
| Hispanic | 85 (25.4) | 81 (24.2) | 75 (22.4) | 72 (21.6) |  |
| White | 122 (36.5) | 141 (42.1) | 189 (56.4) | 176 (52.7) |  |
| Black | 91 (27.2) | 73 (21.8) | 41 (12.2) | 40 (12.0) |  |
| Others | 36 (10.8) | 40 (11.9) | 30 (9.0) | 46 (13.8) |  |
| Marital status, % |  |  |  |  | 0.052 |
| Married / partner | 194 (59.3) | 203 (61.5) | 201 (61.8) | 165 (50.9) |  |
| Widowed/divorced/separated | 80 (24.5) | 76 (23.0) | 84 (25.8) | 100 (30.9) |  |
| Never married | 53 (16.2) | 51 (15.5) | 40 (12.4) | 59 (18.2) |  |
| Household income, $ |  |  |  |  | 0.062 |
| <20000 | 69 (22.0) | 68 (21.0) | 64 (19.9) | 83 (26.3) |  |
| 20000-74999 | 159 (50.8) | 159 (49.1) | 169 (52.6) | 171 (54.3) |  |
| ≥75000 | 85 (27.2) | 97 (29.9) | 88 (27.4) | 61 (19.4) |  |
| Monocyte count, 10^3^/uL | 0.50 [0.40, 0.60] | 0.50 [0.40, 0.60] | 0.50 [0.40, 0.70] | 0.60 [0.50, 0.70] | <0.001 |
| White blood cell count, 10^3^/uL | 5.90 [5.10, 6.90] | 6.70 [5.60, 7.70] | 7.10 [6.10, 8.60] | 8.10 [6.80, 9.60] | <0.001 |
| Red cell distribution width, % | 13.10 [12.50, 13.70] | 13.00 [12.50, 13.70] | 12.90 [12.40, 13.60] | 13.20 [12.60, 14.00] | 0.013 |
| Red blood cell count, million cells/uL | 4.63 [4.24, 4.96] | 4.64 [4.31, 4.91] | 4.61 [4.28, 4.91] | 4.51 [4.17, 4.89] | 0.026 |
| Hemoglobin, g/dL | 14.10 [13.10, 15.00] | 14.00 [13.10, 15.00] | 14.10 [13.20, 15.00] | 13.70 [12.70, 14.80] | 0.001 |
| Hematocrit, % | 41.30 [38.50, 44.30] | 41.10 [38.60, 44.20] | 41.20 [38.70, 44.00] | 40.20 [37.50, 43.40] | 0.004 |
| Mean cell volume, fL | 90.20 [86.50, 93.10] | 89.60 [86.40, 92.70] | 89.80 [87.30, 92.60] | 89.60 [86.50, 92.40] | 0.587 |
| Mean cell hemoglobin, pg | 30.90 [29.40, 32.00] | 30.70 [29.30, 31.70] | 30.80 [29.50, 31.80] | 30.50 [29.10, 31.70] | 0.210 |
| Mean corpuscular hemoglobin concentration, g/dL | 34.10 [33.40, 34.80] | 34.00 [33.30, 34.70] | 34.10 [33.40, 34.80] | 34.00 [33.40, 34.60] | 0.321 |
| Mean platelet volume, fL | 8.40 [7.80, 9.10] | 8.20 [7.60, 8.90] | 8.10 [7.40, 8.80] | 8.10 [7.40, 8.60] | <0.001 |
| Psoriasis status |  |  |  |  | <0.001 |
| No | 238 (71.3) | 245 (73.1) | 220 (65.7) | 189 (56.6) |  |
| Yes | 96 (28.7) | 90 (26.9) | 115 (34.3) | 145 (43.4) |  |
