# Supplementary material for: Association of systemic inflammation index with psoriasis risk and psoriasis severity: A retrospective cohort study of NHANES 2009 to 2014
Source: Medicine (Baltimore). 2024 Feb 23;103(8):e37236. doi: 10.1097/MD.0000000000037236 (PMC11309634; doi:10.1097/MD.0000000000037236)
Supplement: Supplementary file 2 [file medi-103-e37236-s002.docx]

| **Table S2** Basic characteristics of the study population based on psoriasis severity | | | |
| --- | --- | --- | --- |
| Characteristics | No or mild  (n=227) | Moderate to severe  (n=52) | P-value |
| Age, years | 53.00 [39.00, 65.00] | 51.00 [35.00, 61.00] | 0.247 |
| Gender, % |  |  | 0.295 |
| Male | 104 (45.8) | 28 (53.8) |  |
| Female | 123 (54.2) | 24 (46.2) |  |
| BMI, kgm^2^ | 27.90 [25.30, 34.10] | 29.20 [25.60, 33.00] | 0.978 |
| Race, % |  |  | 0.940 |
| Hispanic | 38 (16.7) | 9 (17.3) |  |
| White | 121 (53.3) | 29 (55.8) |  |
| Black | 29 (12.8) | 5 (9.6) |  |
| Others | 39 (17.2) | 9 (17.3) |  |
| Marital status, % |  |  | 0.012 |
| Married / partner | 127 (56.7) | 28 (56.0) |  |
| Widowed/divorced/separated | 64 (28.6) | 7 (14.0) |  |
| Never married | 33 (14.7) | 15 (30.0) |  |
| Household income, $ |  |  | 0.866 |
| <20000 | 50 (22.7) | 10 (20.0) |  |
| 20000-74999 | 111 (50.5) | 25 (50.0) |  |
| ≥75000 | 59 (26.8) | 15 (30.0) |  |
| Monocyte count, 10^3^/uL | 0.50 [0.40, 0.70] | 0.60 [0.50, 0.70] | 0.025 |
| White blood cell count, 10^3^/uL | 6.90 [5.60, 8.70] | 7.10 [5.90, 9.10] | 0.354 |
| Red cell distribution width, % | 13.20 [12.60, 13.90] | 13.50 [12.90, 14.10] | 0.132 |
| Red blood cell count, million cells/uL | 4.62 [4.29, 4.91] | 4.68 [4.29, 4.90] | 0.705 |
| Hemoglobin, g/dL | 13.90 [12.90, 14.90] | 14.10 [12.80, 14.90] | 0.936 |
| Hematocrit, % | 40.80 [38.30, 43.80] | 40.90 [38.00, 44.30] | 0.853 |
| Mean cell volume, fL | 89.80 [86.60, 92.60] | 89.60 [85.70, 91.60] | 0.308 |
| Mean cell hemoglobin, pg | 30.50 [29.30, 31.70] | 30.60 [29.00, 31.50] | 0.320 |
| Mean corpuscular hemoglobin concentration, g/dL | 34.00 [33.30, 34.50] | 34.00 [33.30, 34.60] | 0.991 |
| Mean platelet volume, fL | 8.40 [7.80, 9.20] | 8.40 [7.90, 9.10] | 0.798 |
| Systemic inflammation index | 522.00 [350.93, 755.27] | 609.43 [404.57, 761.17] | 0.380 |
