# Supplementary figures and images for: Association of systemic inflammation index with psoriasis risk and psoriasis severity: A retrospective cohort study of NHANES 2009 to 2014
Source: Medicine (Baltimore). 2024 Feb 23;103(8):e37236. doi: 10.1097/MD.0000000000037236 (PMC11309634; doi:10.1097/MD.0000000000037236)

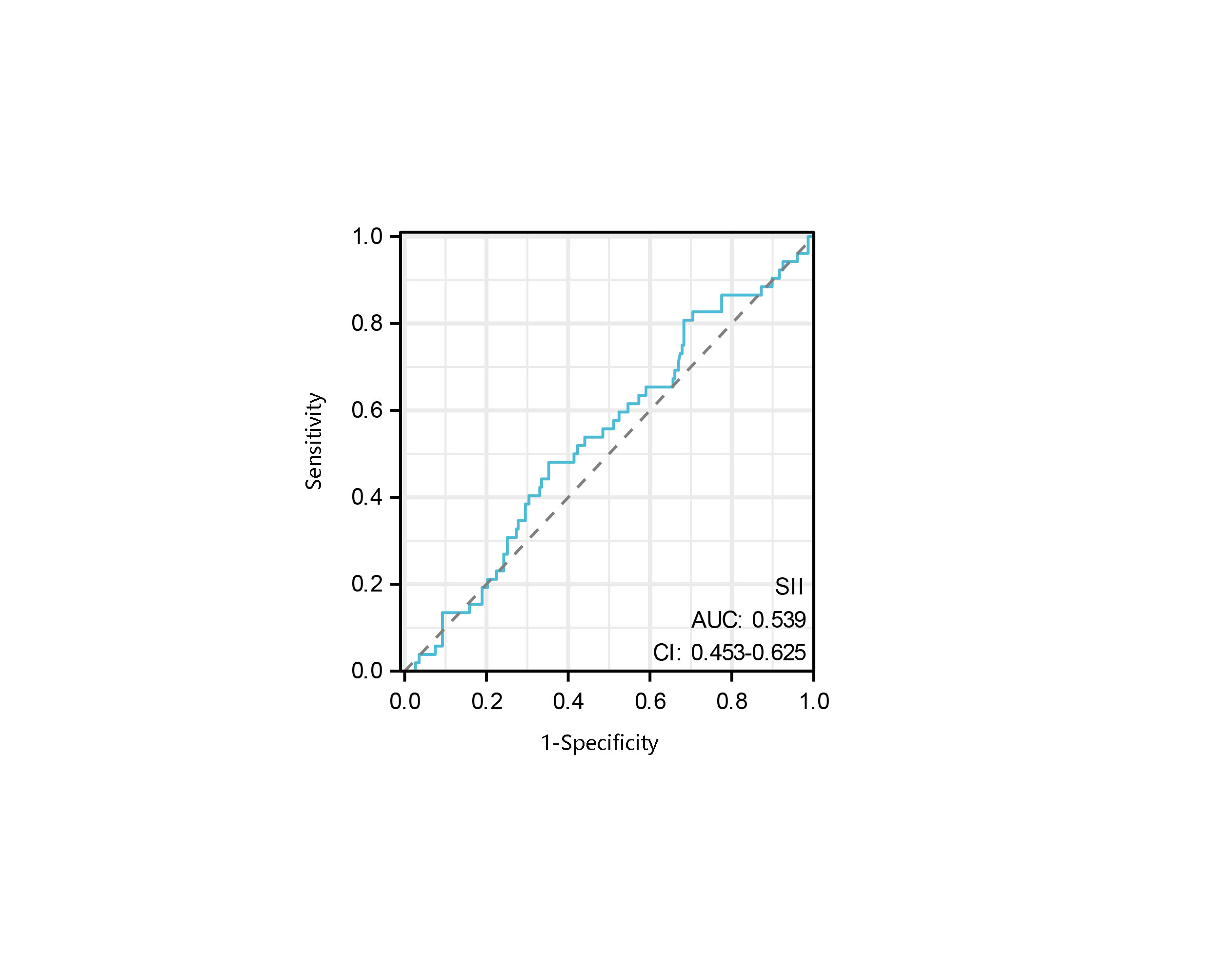

Supplement: Supplementary file 3 [file medi-103-e37236-s003.tif]
